# Long-term exposure to ambient air pollutant and acute exacerbations of chronic obstructive pulmonary disease: a retrospective cohort study in Xinjiang, China

**DOI:** 10.3389/fpubh.2025.1658252

**Published:** 2025-08-27

**Authors:** Zhichuang Lian, Mengxuan Cui, Chao Liu, Ying Chen, Ling Zhang, Bei Wang, Nurula Yakufu, Jiming Xu, Linfeng Li, Xuemei Wei, Julaiti Kelimu

**Affiliations:** ^1^Department of Respiratory and Critical Care Medicine, People’s Hospital of Xinjiang Uygur Autonomous Region, Xinjiang, China; ^2^Yidu Cloud Technology Inc., Beijing, China; ^3^Department of Health Information Management, People’s Hospital of Xinjiang Uygur Autonomous Region, Xinjiang, China; ^4^Department of Automation, Tsinghua University, Beijing, China

**Keywords:** ozone exposure, chronic obstructive pulmonary disease (COPD), vulnerable population, air pollutants, exacerbation

## Abstract

**Introduction:**

While short-term ambient air pollution is implicated in chronic obstructive pulmonary disease (COPD) exacerbations, evidence for chronic exposure remains limited, particularly in vulnerable subgroups. This study evaluates longitudinal associations between major air pollutants and acute exacerbations (AEs), while identifying high-risk demographic and clinical subgroups.

**Methods:**

We analyzed 660 COPD patients from People’s Hospital of Xinjiang (2020–2023). Annual average concentrations of PM_2.5_, PM_10_, O_3_, NO_2_, SO_2_, and CO were geocoded to residential addresses. Single-pollutant logistic regression models adjusted for 12 clinical/sociodemographic confounders assessed AE risks, with robustness verified by two-pollutant sensitivity analyzes. Stratified analyzes examined effect modification across 10 key parameters including disease severity, smoking status, comorbidities, and sociodemographic characteristics.

**Results:**

Long-term ozone exposure demonstrated significant AE risk elevation (OR = 1.007, *p* = 0.046). This association was confirmed to be robust in two-pollutant models. Stratified analyzes revealed amplified effects in males (OR = 1.009, *p* = 0.046), those aged over 65 years (OR = 1.012, *p* = 0.014), Han ethnicity (OR = 1.019, *p* = 0.003), those with prior-year AEs (OR = 1.008, *p* = 0.048), and non-asthmatics (OR = 1.014, *p* = 0.009).

**Conclusion:**

This study establishes chronic ozone exposure as an emerging environmental determinant of COPD exacerbations, with disproportionate impacts on vulnerable subgroups. Our findings demand urgent integration of ozone mitigation into national respiratory health strategies and precision public health approaches to address environmental health inequities.

## 1 Introduction

Chronic obstructive pulmonary disease (COPD) is a progressive respiratory disorder characterized by persistent airflow limitation (1). It represents a significant global health challenge, ranking as the third leading cause of death worldwide (2–4) and in China (5). Acute exacerbations (AEs) represent sudden episodes of worsening symptoms in COPD patients, marked by increased dyspnea, cough, and sputum production (6, 7). These exacerbations have been associated with accelerated decline in lung function, increased rates of hospitalizations, and higher mortality (8–10), underscoring the need for effective management strategies.

The causes of AEs are multifactorial, including viral and bacterial infections (11–13) as well as smoking (14). In recent years, air pollution and its direct health impacts has emerged as a significant concern in both general medicine (15, 16) and respiratory medicine (17). Ambient air pollutants, such as particulate matter (PM_2.5_), inhalable particulate matter (PM_10_), nitrogen dioxide (NO_2_), carbon monoxide (CO), sulfur dioxide (SO_2_), and ozone (O_3_), have been implicated in triggering exacerbations (18–20). Biological evidences suggest that these pollutants can induce inflammation (21), oxidative stress (22, 23), and systemic effects, thereby exacerbating the underlying pathophysiology of COPD (13, 24).

Growing evidence suggests ambient air pollution contributes to COPD exacerbations, yet critical knowledge gaps persist regarding long-term exposure effects and population-specific susceptibility (25–29). Most previous studies have focused on short-term effects of these air pollutants on AEs (25, 26), while longitudinal associations spanning months to years remain understudied. Furthermore, due to data limitations, few studies have examined effect modification by age or sex, and very few have explored potential modifications by disease severity, lifestyle factors, or comorbidities (19). Notably, data indicated a significant improvement in air quality in recent years, while O_3_ had emerged as a significant risk factor in China (18), necessitating updated interventions based on real-world evidence.

To address these limitations, we conducted a cohort study to investigate the long-term effects of air pollutant exposure on AEs of COPD in Xinjiang—a multi-ethnic region. Furthermore, we also aimed to identify potentially susceptible sub-populations using extensive individual-level data. These results will facilitate targeted environmental interventions and personalized prevention to mitigate the impact of air pollution on COPD outcomes.

## 2 Materials and methods

### 2.1 Study design and study population

This is a retrospective study performed at the People’s Hospital of Xinjiang Uygur Autonomous Region (PHXUAR) in Urumqi, Xinjiang, China.

**Figure 1:**
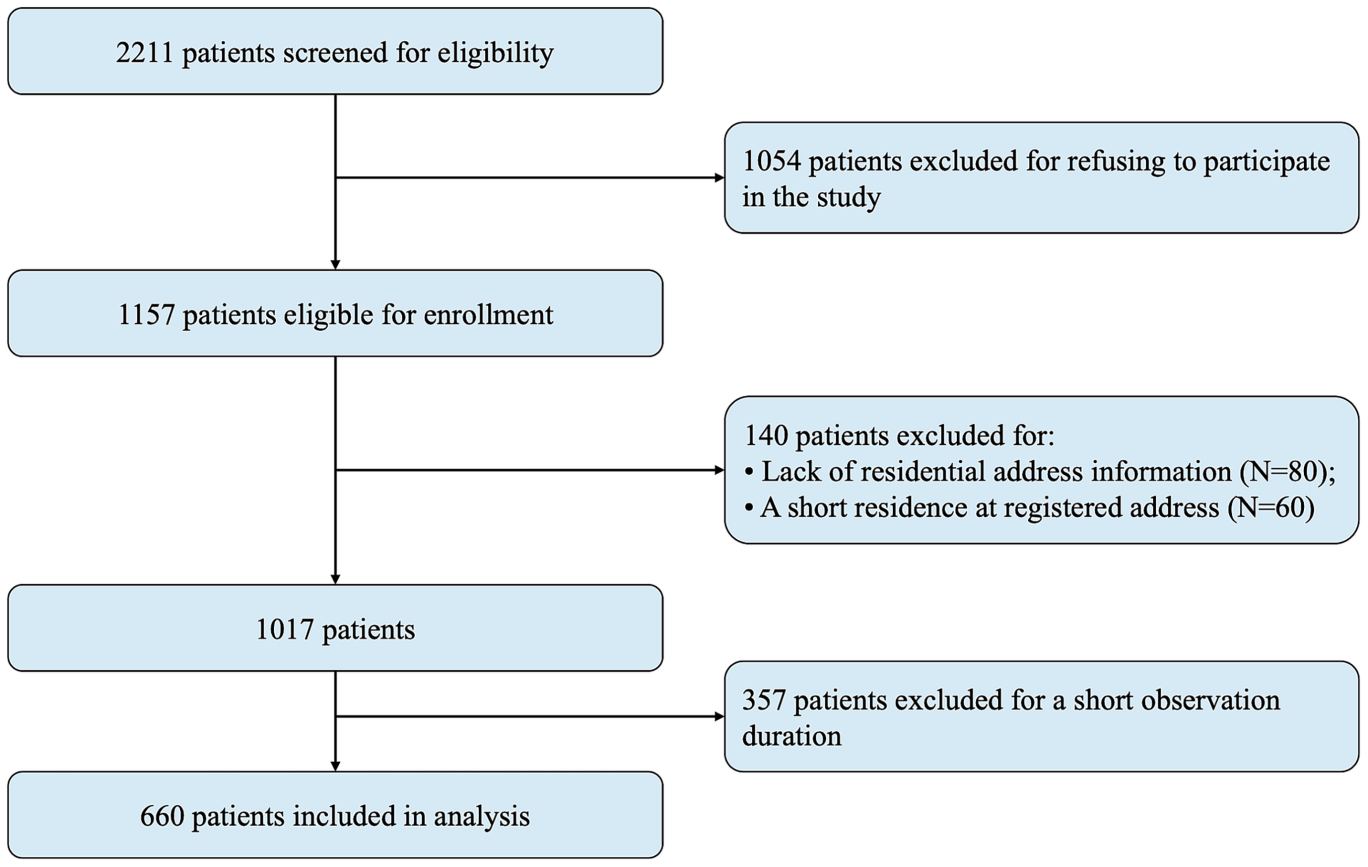
Flow chart of the study.

The study period spanned from January 1, 2020, to December 31, 2023. Patients with a post-bronchodilator FEV1/FVC ratio <0.7 (30), as determined by spirometry, were initially considered for inclusion. The baseline was defined as the date of the first occurrence of FEV1/FVC < 0.7 at the hospital. The baseline period was defined as the three-month interval immediately before and after this date, during which baseline characteristics were collected. Participants were required to have had at least one in-person hospital visit after the end of the baseline period. Exclusion criteria included: (1) age under 18 years, (2) missing outcome, (3) absence of residential address information or a transient residence at the registered address, and (4) a short observation duration (<1 year). Consequently, 660 participants were deemed eligible for analysis. A flow chart detailing the inclusion and exclusion process was provided in Figure 1.

### 2.2 Outcome

AEs were identified through participants’ medical records at the hospital. The diagnosis of an AE of COPD was established by physicians based on a combination of patients’ clinical signs and symptoms, spirometry results, chest imaging findings, and other pertinent clinical data. The outcome in this study was defined as occurrence of AE within the first year after baseline period.

### 2.3 Assessment of air pollutants

Air pollutant data for this study were obtained from the National Urban Air Quality Real-time Publishing Platform^1^, which compiles hourly measurements from all fixed-site monitoring stations across China. These data were published by the China National Environmental Monitoring Center under the administration of China’s Ministry of Ecology and Environment. In this study, missing data for specific hours were estimated by calculating the average of the data from the immediately preceding and succeeding hours.

For each participant in our study, we extracted their permanent addresses in Xinjiang and geocoded these addresses to obtain longitude and latitude coordinates. Subsequently, we matched each participant’s residential address to the nearest fixed-site monitoring station to retrieve the most relevant pollution data. The distance between each participant’s location and the nearest monitoring station was calculated using the “geodesic” function from “geopy” library in Python (Version 3.9). We then calculated the average concentrations of PM_2.5_, PM_10_, O_3_, NO_2_, SO_2_, and CO during each participant’s respective baseline period and used these averages as a proxy for personal exposure in this study. For participants without a specific address on record, the average pollution levels for their respective city were utilized to estimate their exposure.

### 2.4 Confounders

We systematically collected characteristics from medical records that have been previously associated with AEs, including GOLD grade and severity, age, gender, ethnicity, occupation, education level, marital status, residential setting, tobacco smoking history, body mass index (BMI), comorbid asthma, hypertension, diabetes, dyslipidemia, cardiovascular disease, and a history of AEs in the past year.

Based on the GOLD spirometry classification (30), the severity of COPD was categorized as follows: FEV1 ≥ 80% of predicted value was designated as GOLD 1 (mild), 50% ≤ FEV1 < 80% of predicted value as GOLD 2 (moderate), 30% ≤ FEV1 < 50% of predicted value as GOLD 3 (severe), and FEV1 < 30% of predicted value as GOLD 4 (very severe). BMI was categorized as follows (31): a BMI of less than 18.5 was classified as underweight, 18.5 to 23 as normal weight, and greater than 23 as obese.

Socioeconomic factors, including occupation, education level, and marital status, were considered in their potential association with AEs of COPD. However, data on occupation and education level were missing for over 30% of the participants, and the vast majority (over 95%) were married, which limited the contribution of these factors in the analysis. Consequently, these socioeconomic variables were not included as confounders in the study.

### 2.5 Statistical analysis

Baseline characteristics of continuous variables were reported as means (standard deviations), and categorical variables were reported as counts (percentages). Air pollutant concentrations were described by maximum values, minimum values, and quartile values. Differences across cohorts were compared using T, Mann–Whitney U, or χ^2^ tests, as appropriate. The Kolmogorov–Smirnov test was used to assess the normality of the variable distributions.

In the initial analysis, the occurrence of AEs within the first year was examined as the outcome variable in univariate logistic regression models. By this approach, associations between AEs and all variables, including air pollutants and the confounders, were explored. Subsequently, we developed single-pollutant models for each pollutant. Adjusting for age, gender, ethnicity, residential setting, smoking status, and BMI, as well as asthma, diabetes, dyslipidemia, CVD, history of AEs in the past year, and FEV1% predicted at baseline, six multivariable logistic regression models were built to describe the association of each air pollutant with incidence of AEs. GOLD grade, FEV1/FVC, season of baseline, and hypertension were removed for multicollinearity. Occupation and education level were removed for high missing rate. Risk estimates were expressed as odds ratios (ORs), with 95% confidence intervals (CIs) and corresponding *p*-values. The multivariable regression model was constructed using the “glm” function.

To examine the robustness of observed associations, sensitivity analyzes were performed. We extended our analysis beyond single-pollutant models by fitting two-pollutant models for each pair of the six pollutants, with the aim to control for potential confounding effects due to co-exposures. The pair of PM_2.5_ and PM_10_ were excluded as PM_2.5_ is inherently a component of PM_10_ measurements.

Utilizing the extensive individual-level features, a series of stratified analyzes were conducted to elucidate the specific associations between air pollution and AEs of COPD in subgroups. These analyzes were stratified by and disease severity (GOLD grade 1 vs. grade 2/3), age (≤65 years vs. >65 years), gender (male vs. female), ethnicity (Uyghur vs. Han), residential setting (rural vs. urban), smoking status (no smoking history vs. previous or current smoking history), frequency of AEs of COPD in the past year (0 vs. ≥1), a history of CVD (yes vs. no), a history of asthma (yes vs. no), and season of baseline (spring vs. winter).

All statistical analyzes were performed using R-Studio version 4.1.2 (R Foundation for Statistical Computing, Vienna, Austria). Two-sided *p*-values of less than 0.05 were considered statistically significant.

## 3 Results

### 3.1 Study population

As shown in Figure 1, of the initial 2,211 patients diagnosed with COPD based on an FEV1/FVC ratio of less than 0.7, all were above 18 years of age. Exclusion criteria led to the removal of 1,054 patients who did not report their outcome of AEs in the following year. Furthermore, 140 participants were excluded due to missing residential addresses or not residing for at least 3 years at their registered addresses. Additionally, 357 individuals who were observed for a short duration were also excluded. Consequently, a total of 660 patients were included in the statistical analysis.

Table 1 presents the baseline characteristics of the eligible participants. The FEV1% predicted values were nearly normally distributed, with a median of 102.66 and a mean of 102.73. The majority of participants were categorized as having mild COPD. Notably, those in the AE group exhibited significantly lower FEV1% predicted and FEV1/FVC ratios at baseline. The majority of participants were admitted during the winter months, accounting for 61.8% of the total admissions, while the remaining 38.2% were admitted in the spring. The average age was 64.8 ± 10.5 years, spanning from 30 to 88 years. The majority of participants were male (64.1%). Ethnic distribution was 54.1% Uyghur, 36.8% Han Chinese, and 9.1% other ethnicities. Urban residence was reported by 62.7% of the participants. 39.5% had a smoking history. 6.6% of participants were classified as low body weight. Concurrent COPD and asthma diagnoses were present in 56.4% of the participants. Additionally, the prevalence of hypertension, diabetes, dyslipidemia, and CVD was 64.4, 30.5, 20.0, and 65.3%, respectively. Regarding history of AEs of COPD, 263 (39.8%) participants did so once, and 195 (29.5%) had two or more within the previous year.

**Table 1:** Baseline characteristics of participants with and without acute exacerbations in the following year.

	All patients (*N* = 660)	Outcome = 0 (*N* = 487)	Outcome = 1 (*N* = 173)	*P*
Spirometry				
GOLD grade and severity (%)				0.519
Mild – GOLD 1	545 (82.6)	405 (83.2)	140 (80.9)	
Moderate – GOLD 2	113 (17.1)	80 (16.4)	33 (19.1)	
Severe – GOLD 3	2 (0.3)	2 (0.4)	0 (0.0)	
FEV1% predicted (mean (SD))	102.73 (23.63)	103.86 (23.88)	99.55 (22.69)	0.039
FEV1/FVC (mean (SD))	60.01 (7.73)	60.47 (7.46)	58.71 (8.35)	0.01
Season of spirometry				
Season				0.777
Spring (March–May)	252 (38.2)	188 (38.6)	64 (37.0)	
Winter (December–February)	408 (61.8)	299 (61.4)	109 (63.0)	
Demographic factors				
Age (mean (SD))	64.81 (10.53)	64.98 (10.67)	64.34 (10.14)	0.496
Gender (%)				0.765
Female	237 (35.9)	177 (36.3)	60 (34.7)	
Male	423 (64.1)	310 (63.7)	113 (65.3)	
Ethnicity (%)				0.598
Uyghur	357 (54.1)	266 (54.6)	91 (52.6)	
Han	243 (36.8)	180 (37.0)	63 (36.4)	
Other	60 (9.1)	41 (8.4)	19 (11.0)	
Socioeconomic factors				
Occupation (%)				0.023
Retired	304 (46.1)	213 (43.7)	91 (52.6)	
Farmer	45 (6.8)	39 (8.0)	6 (3.5)	
Worker	28 (4.2)	26 (5.3)	2 (1.2)	
Other	66 (10.0)	48 (9.9)	18 (10.4)	
Unknown	217 (32.9)	161 (33.1)	56 (32.4)	
Education level (%)				0.572
Primary or no education	87 (13.2)	67 (13.8)	20 (11.6)	
Middle or high school	189 (28.6)	139 (28.5)	50 (28.9)	
College or higher	138 (20.9)	96 (19.7)	42 (24.3)	
Unknown	246 (37.3)	185 (38.0)	61 (35.3)	
Marriage status (%)				0.612
Married	631 (95.6)	464 (95.3)	167 (96.5)	
Never married	2 (0.3)	2 (0.4)	0 (0.0)	
Divorced	3 (0.5)	3 (0.6)	0 (0.0)	
Widowed	24 (3.6)	18 (3.7)	6 (3.5)	
Residential setting (%)				0.017
Rural	246 (37.3)	195 (40.0)	51 (29.5)	
Urban	414 (62.7)	292 (60.0)	122 (70.5)	
Lifestyle factors				
Smoking (%)				0.844
Never smoking	399 (60.5)	296 (60.8)	103 (59.5)	
Ever smoking	261 (39.5)	191 (39.2)	70 (40.5)	
Clinical factors				
BMI (%)				0.184
Under-weight	24 (3.6)	16 (3.3)	8 (4.6)	
Normal	137 (20.8)	109 (22.4)	28 (16.2)	
Over-weight	499 (75.6)	362 (74.3)	137 (79.2)	
Asthma (%)	372 (56.4)	273 (56.1)	99 (57.2)	0.86
Hypertension (%)	425 (64.4)	312 (64.1)	113 (65.3)	0.839
Diabetes (%)	201 (30.5)	145 (29.8)	56 (32.4)	0.588
Dyslipidemia (%)	132 (20.0)	100 (20.5)	32 (18.5)	0.642
CVD (%)	431 (65.3)	310 (63.7)	121 (69.9)	0.162
History of AEs of COPD (%)	458 (69.4)	323 (66.3)	135 (78.0)	0.006

Data are presented as No. (%) or mean (SD). CVD, cardiovascular disease; AEs, acute exacerbations; COPD, chronic obstructive pulmonary disease; History of AEs of COPD: in previous 1 year.

We preformed exploratory analysis on the associations between these factors and AEs (Supplementary Table S1). Urban residency and a history of AEs in the previous year were significantly associated with AEs in the univariate analysis (ORs of 1.091 [95% CI 1.018–1.169, *p* = 0.014] and 1.113 [95% CI 1.035–1.196, *p* = 0.004], respectively).

### 3.2 Exposure to air pollutants

**Figure 2:**
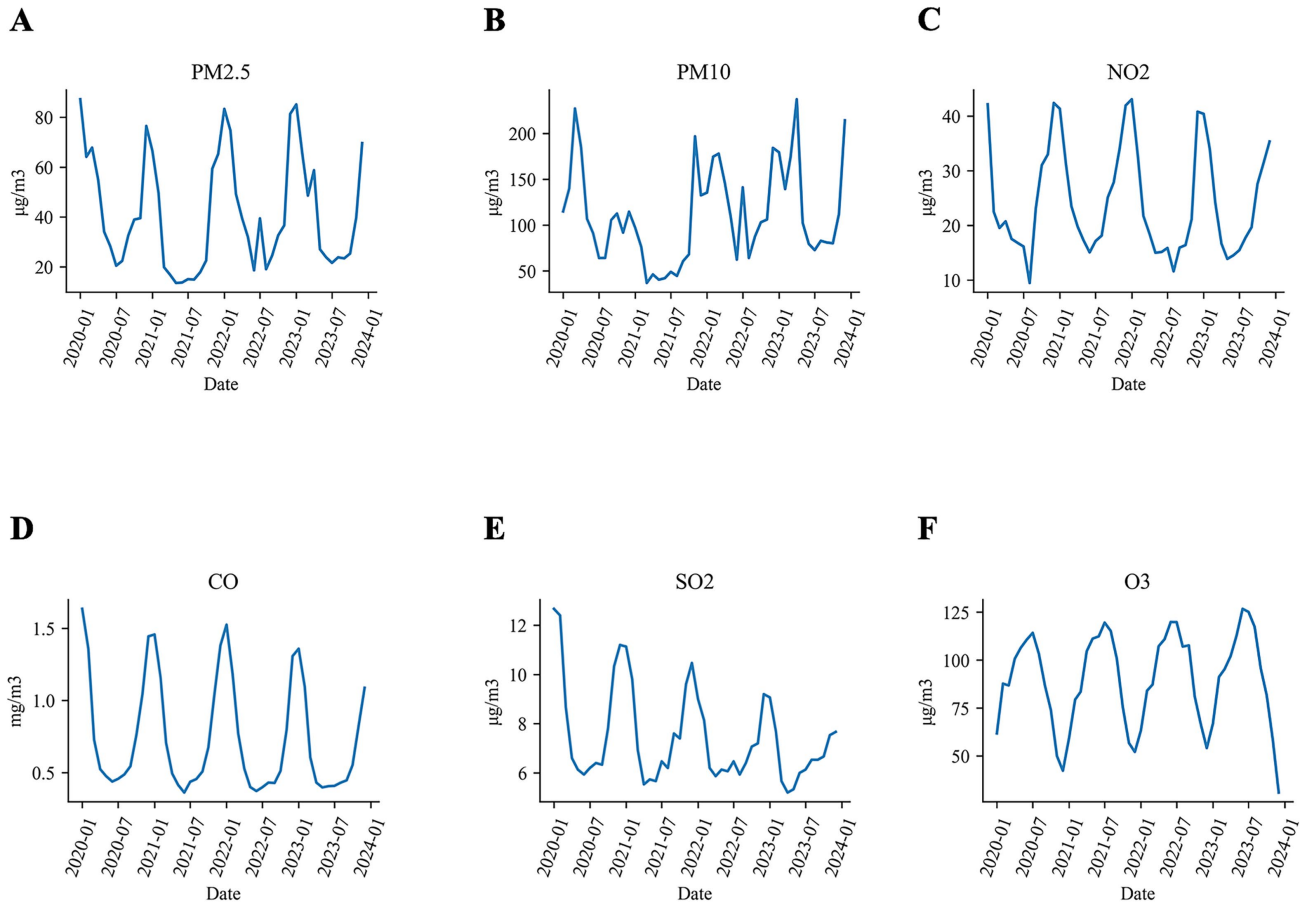
The average concentrations of air pollutants in Xinjiang from 2020 to 2023 in Xinjiang **(A)** PM_2.5_, **(B)** PM_10_, **(C)** NO_2_, **(D)** CO, **(E)** SO_2_, and **(F)** O_3_.

Figure 2 illustrates the average concentrations of air pollutants across fifteen cities in Xinjiang from 2010 to 2023. Over this period, the annual mean concentration of NO_2_ declined by 13% (from 27.9 μg/m^3^ in 2017 to 24.2 μg/m^3^ in 2023), CO concentration declined by 26% (from 0.91 mg/m^3^ to 0.67 mg/m^3^), and SO_2_ declined by 27% (from 9.1 μg/m^3^ to 6.7 μg/m^3^). Whereas O_3_ concentration increased by 5.9% (from 86.9 μg/m^3^ to 92.0 μg/m^3^). Regarding particulate matter, both PM_2.5_ and PM_10_ concentrations remained relatively stable, with the exception of the year 2021 when COVID-19 restrictions reduced industrial activities, leading to a temporary dip in concentration levels.

The baseline dates for the cohort ranged from 2020 to 2023. We defined the baseline period as a six-month interval centered by the baseline date, during which we collected data on air pollutants. Table 2 summarizes the six-month average concentrations of air pollutants to which the cohort was exposed. The median levels of these pollutants were as follows: 45.6 μg/m^3^ for PM_2.5_, 84.3 μg/m^3^ for PM_10_, 33.7 μg/m^3^ for NO_2_, 0.9 mg/m^3^ for CO, 7.9 μg/m^3^ for SO_2_, and 87.9 μg/m^3^ for O_3_. The distribution of these pollutants by study outcome was detailed in Supplementary Figure S1.

**Table 2:** Distributions of ambient air pollutant exposure for participants.

	PM2.5 (μg/m^3^)	PM10 (μg/m^3^)	NO_2_ (μg/m^3^)	CO (mg/m^3^)	SO_2_ (μg/m^3^)	O_3_ (μg/m^3^)
Overall						
Minimum	8.62	14.97	9.82	0.42	3.15	75.59
Q25	42.30	75.05	27.31	0.77	7.13	83.64
Median	45.62	84.33	33.67	0.90	7.85	87.91
Q75	51.50	147.62	36.95	1.01	8.38	93.28
Maximum	90.18	301.49	40.77	1.44	16.92	99.31

Q25, the 25th percentile; Q75, the 75th percentile; PM2.5, particulate matter ≤ 2.5 μm in aerodynamic diameter; PM10, particulate matter ≤ 10 μm in aerodynamic diameter; NO2, nitrogen dioxide; CO, carbon monoxide; SO2, sulfur dioxide; O3, ozone.

### 3.3 Associations of AEs with air pollutants

The relationships between each air pollutant and the occurrence of at least one AE during the first year of follow-up are detailed in Table 3. In the initial univariate logistic regression analysis, no significant associations were observed between air pollutant exposure and the occurrence of AEs. After adjusting for confounders in the single-pollutant multivariable analysis, exposure to O_3_ was found to be associated with the risk of AEs (OR = 1.007 [95% CI 1.000–1.013], *p* = 0.046). By contrast, exposure to PM_2.5_, PM_10_, NO_2_, SO_2_, and CO showed no association with AEs in the entire cohort, with ORs [95% CI] as 1.000 [0.997–1.002], 1.000 [1.000–1.001], 0.996 [0.991–1.000], 0.902 [0.746–1.091], and 0.997 [0.982–1.012], respectively.

**Table 3:** Unadjusted and adjusted ORs for the acute exacerbations of COPD associated with each unit increase in the concentrations of air pollutant in single-pollutant models.

	Unadjusted Model		Single-pollutant Multivariable Model	
	OR [95% CI]	*P*	OR [95% CI]	*P*
PM2.5	0.999 [0.997–1.001]	0.573	1 [0.997–1.002]	0.689
PM10	1 [0.999–1]	0.804	1 [1–1.001]	0.602
NO2	0.998 [0.994–1.002]	0.438	0.996 [0.991–1]	0.059
CO	0.92 [0.763–1.109]	0.382	0.902 [0.746–1.091]	0.290
SO2	0.994 [0.979–1.009]	0.416	0.997 [0.982–1.012]	0.695
O3	1.003 [0.997–1.009]	0.317	1.007 [1–1.013]	0.046

Single-pollutant model adjusted for FEV1% predicted at baseline, age, gender, ethnicity (Han, Uyghur, and other), residential setting (urban and rural), smoking status (ever smoking or not), BMI (under-weight, normal, and over-weight), asthma, CVD, and history of AEs of COPD in previous 1 year. GOLD grade, FEV1/FVC, hypertension, and season of baseline were removed for multicollinearity. Occupation and education level were removed for high missing rate. PM2.5, particulate matter ≤2.5 μm in aerodynamic diameter; PM10, particulate matter ≤ 10 μm in aerodynamic diameter; NO2, nitrogen dioxide; CO, carbon monoxide; SO2, sulfur dioxide; O3, ozone.

### 3.4 Sensitivity analysis

**Figure 3:**
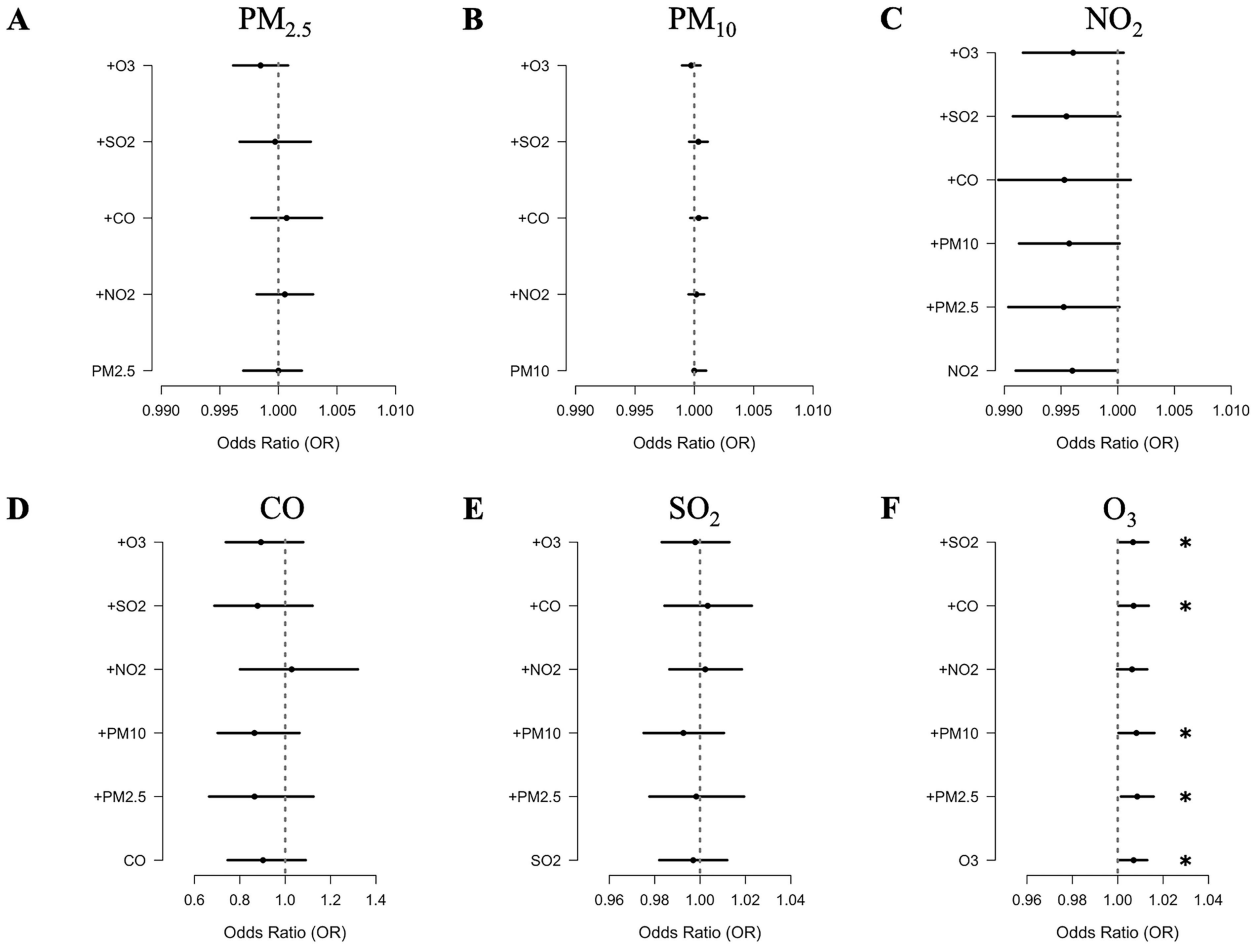
Adjusted ORs for the acute exacerbations of COPD associated with each unit increase in the concentrations of **(A)** PM_2.5_, **(B)** PM_10_, **(C)** NO_2_, **(D)** CO, **(E)** SO_2_, and **(F)** O_3_ in two-pollutant models.

The results of two-pollutant models are presented in Figure 3 and Supplementary Table S2. We found that the associations between all pollutant factors and AEs in the following year were robust after adjusting for co-exposure. For O_3_, the associations were consistently significant in all the models except for adjustments for NO_2_, with ORs of 1.006 [95% CI 1–1.013, *p* = 0.065]. For PM_2.5_, PM_10_, NO_2_, SO_2_, and CO, the associations remained insignificant, regardless of adjustments for any other air pollutant.

### 3.5 Stratified analyzes

**Figure 4:**
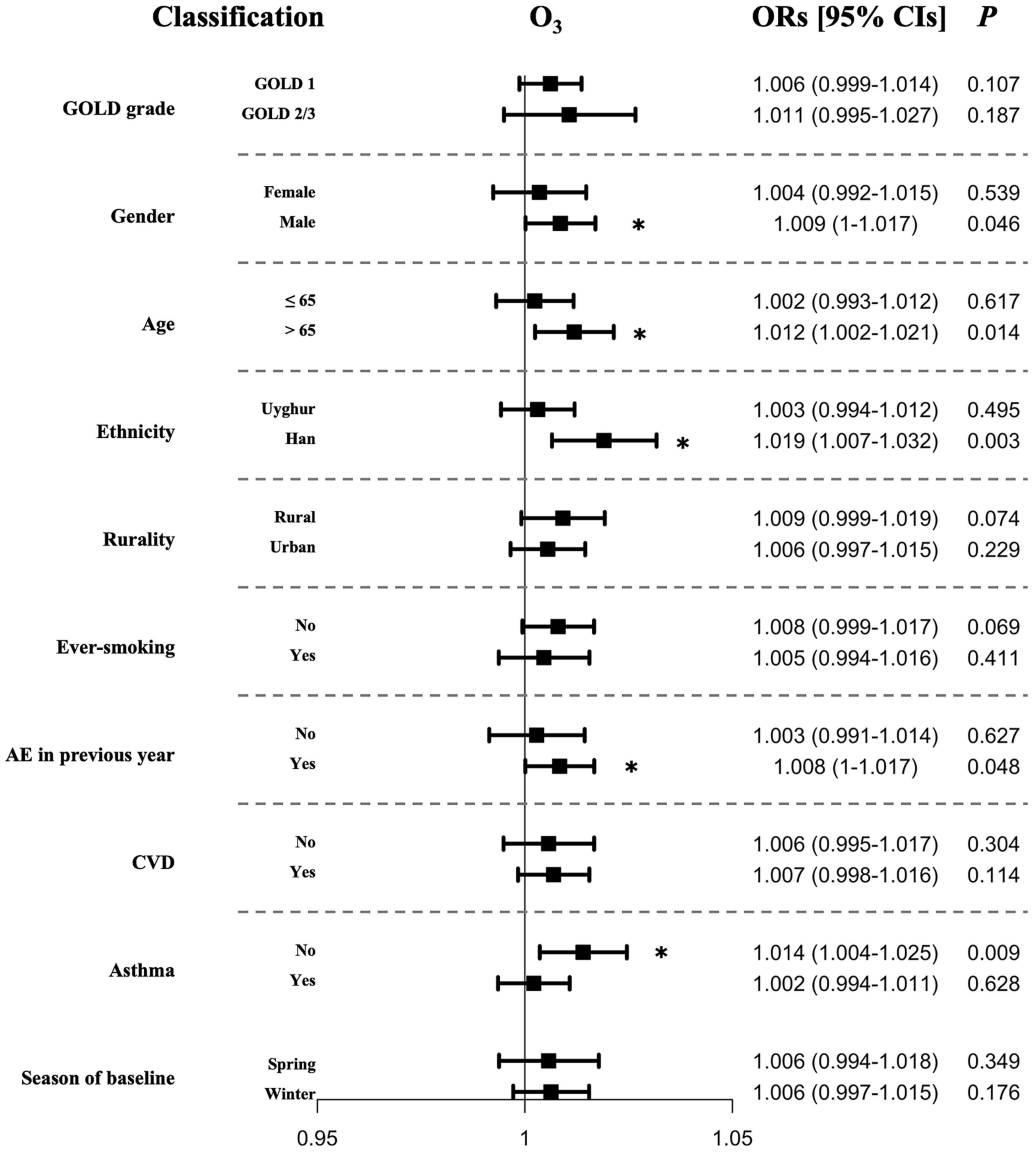
Adjusted ORs for the acute exacerbations of COPD associated with each unit increase in the concentrations in O_3_ in stratified analyzes.

Subgroup analyzes were conducted to determine whether adjusted associations between AEs of COPD and O_3_ varied across different individual characteristics. By this approach, we identified specific sub-populations that were more susceptible to the effects of O_3_ (Figure 4). Our findings revealed that the associations of O_3_ with AEs of COPD were relatively larger in male patients (OR of 1.009 [95% CI 1–1.017], *p* = 0.046), those aged over 65 years (OR of 1.012 [95% CI 1.002–1.021], *p* = 0.014), those of Han ethnicity (OR of 1.019 [95% CI 1.007–1.032], *p* = 0.003), those with a history of AEs in the previous year (OR of 1.008 [95%CI 1–1.017], *p* = 0.048), and those without a concurrent diagnosis of asthma (OR of 1.014 [95%CI 1.004–1.025], *p* = 0.009). No differences were observed between subgroups defined by varying disease severity, residential settings, smoking history, or the presence of CVD history. Furthermore, for PM_2.5_, PM_10_, NO_2_, SO_2_, and CO, the associations remained insignificant in sub-populations analyzed (Supplementary Table S3).

## 4 Discussion

In this study, we investigated the chronic effect of air pollutant exposure on AEs of COPD. Our findings indicated that chronic exposure to O_3_ was associated with an increased risk of AEs, which was shown to be robust in sensitivity analyzes. In contrast, the associations with PM_2.5_, PM_10_, NO_2_, SO_2_, and CO were insignificant in both single- and two-pollutant models. Utilizing a well-characterized cohort of COPD patients, we further provided novel insights into populations particularly susceptible to O_3_ exposure. Specifically, males over 65 years old, individuals of Han ethnicity, those with a history of AEs, and patients without comorbid of asthma might be more vulnerable.

The association between ozone and AEs of COPD has yield inconclusive results in previous studies (25–29). Several studies based on the SPIROMICS AIR cohort have reported adverse effect of long-term ambient ozone on COPD exacerbation, thereby reinforcing our findings (28, 29). Conversely, numerous literatures have reported insignificant or even positive associations of ozone exposure on AEs (25–27). For instance, researches from Germany (27) and Korea (26) have suggested that short-term ozone exposure was not correlate with increased hospital visits for AEs. Additionally, a study conducted in Canada (25) found a positive association between short-term ambient ozone exposure and AEs. These discrepancies imply that the effects of ozone on AEs in COPD may vary depending on the duration of exposure, with short- and long-term effects potentially diverging in their impact on disease exacerbation. Such observations underscore the complexity of understanding the relationship between air pollution and COPD outcomes, and also highlight the need for further research to elucidate the temporal dynamics of ozone exposure and its effects on respiratory health.

Plenty of biological studies support the ozone effect on AEs in COPD (32–36). Firstly, exposure to ozone induces airway hyperresponsiveness (32), which is a hallmark of AEs in COPD. Secondly, as an oxidant, ozone generates reactive oxygen species in airways, leading to oxidative stress and inflammation, which potentially triggering onset of AEs (33, 34). Thirdly, ozone exposure can disrupt tight junctions in the epithelial cells, increasing susceptibility to infections (35, 36).

Identifying vulnerable populations is of great importance for managing AEs. Consistent with prior studies (37, 38), we found that men aged over 65 years were more susceptible to ozone exposure. Additionally, we confirmed previous findings (39, 40) that patients with a history of exacerbations were more vulnerable to ozone exposure. Notably, we identified distinct vulnerable populations. Patients of Han ethnicity were more vulnerable to ozone exposure than those of Uyghur ethnicity. We speculate that these findings might be attributed to unmeasured confounders, especially varying levels in disease awareness, self-management, and education between these ethnic groups. Factors such as anatomical differences in airway structure, dietary habits, and cultural practices related to physical activity between Han and Uyghur ethnic groups could also play a role in influencing COPD outcomes. Furthermore, among patients with COPD, those without asthma were more vulnerable than those with asthma. Except for disease awareness levels, one possible explanation is that asthmatic COPD patients may already have a more robust immune response to irritants, which could mitigate some harmful effects compared to non-asthmatic COPD patients.

Seasonality has been recognized as a potential factor influencing the severity and frequency of COPD exacerbations (20). However, the role of seasonality in COPD exacerbations may vary across different populations and geographic regions. In our study, we adjusted for seasonality, but no moderating or influencing effect of season on the primary findings was observed.

Although several previous investigations have implicated negative effects of PM_2.5_ and/or PM_10_ in the exacerbation of COPD (25, 41), our data failed to substantiate these associations. Instead, our results aligned closely with those of Evangelopoulos D et al. (20), who reported that gaseous pollutants (NO_2_, O_3_, NO, and CO) adversely affect respiratory health, while particulate pollutants did not, over an average follow-up period of 128 days. Notably, they utilized personal portable monitors to measure the total exposure, contrasting with our ambient exposure approach using data from fixed-site station. Despite this difference, both our study and theirs demonstrated a close alignment in the duration of exposure and the associations between ozone and AEs.

A Study from Guangdong Province, China (18), indicated a significant improvement in air quality in recent years, with substantial reductions in PM_2.5_, PM_10_, and SO_2_, while O_3_ had emerged as a significant risk factor in the region. Consistent with these trends, we observed a marked downward in NO_2_, CO, and SO_2_, alongside an increase in O_3_ level. Both the data from Guangdong Province and our study underscored the need for focused strategies to mitigate ozone pollution, highlighting its emergence as a key environmental threat to respiratory health.

Our study has several limitations. Firstly, the study design may introduce potential biases. The patient cohort was comprised exclusively of patients seeking hospital care, which may not be representative of the broader COPD population, thus introducing a selection bias. The cohort was drawn from a single hospital and was relatively small, with the majority of participants having mild to moderate COPD, which also indicted selection bias and limited the statistical power of the study. Moreover, AE events were identified based on hospital visits dates rather than precise onset times, potentially introducing temporal bias in outcomes assessments and possibly missing events that occurred outside medical settings. Secondly, using ambient air pollution exposure as a proxy for personal exposure lack precision. Data from fixed monitoring locations only measure ambient air pollution exposure and may not accurately reflect total personal exposure (42, 43), especially for those spent significant time indoors. Future studies should consider using personal portable monitors to obtain more reliable exposure data. Thirdly, although we included numerous confounders, the nature of the cohort study design made it vulnerable to influences from unmeasured confounders. Our models lack of complete control for climatic variables (temperature, humidity) or geographical factors (altitude, indoor air quality), socioeconomic status and lifestyle factors, which may confound pollution-exacerbation relationships. Particularly in Xinjiang’s extreme continental climate, future studies should incorporate high-resolution meteorological data and indoor pollution monitoring.

## 5 Conclusion

In conclusion, this study establishes chronic ozone exposure as an emerging environmental determinant of COPD exacerbations, revealing disproportionate impacts on vulnerable subgroups in China’s evolving air pollution landscape. Our findings demand urgent integration of ozone mitigation into national respiratory health strategies, prioritizing at-risk populations while advancing precision public health. Beyond immediate clinical implications, this work underscores the imperative to reconcile air quality progress with persistent environmental health inequities.

## Data Availability

The demonstration data are publicly available on GitHub at https://github.com/CMXyiduyun/AECOPD. Further inquiries can be directed to the corresponding author.

## Footnotes

^1^ https://air.cnemc.cn:18007/
