# Supplementary material for: Long-term exposure to ambient air pollutant and acute exacerbations of chronic obstructive pulmonary disease: a retrospective cohort study in Xinjiang, China
Source: Front Public Health. 2025 Aug 27;13:1658252. doi: 10.3389/fpubh.2025.1658252 (PMC12421444; doi:10.3389/fpubh.2025.1658252)
Supplement: Supplementary file 1 [file Data_Sheet_1.docx]

**Supplement**

**Table S1.** ORs for the covariates

|  | **OR [95% CI]** | ***P* value** |
| --- | --- | --- |
| **Spirometry** |  |  |
| GOLD grade and severity |  |  |
| GOLD 1 | reference |  |
| GOLD 2 | 1.036 [0.947-1.132] | 0.440 |
| GOLD 3 | 0.773 [0.42-1.426] | 0.411 |
| FEV1% predicted | 0.999 [0.997-1] | 0.039 |
| FEV1/FVC | 0.994 [0.99-0.999] | 0.010 |
| **Demographic factors** |  |  |
| Age | 0.999 [0.996-1.002] | 0.496 |
| Gender |  |  |
| Female | reference |  |
| Male | 1.014 [0.945-1.088] | 0.696 |
| Ethnicity |  |  |
| Han | reference |  |
| Uyghur | 0.996 [0.927-1.07] | 0.905 |
| Other | 1.059 [0.935-1.199] | 0.366 |
| **Lifestyle factors** |  |  |
| Residential setting |  |  |
| Rural | reference |  |
| Urban | 1.091 [1.018-1.169] | 0.014 |
| Smoking |  |  |
| Never smoker | reference |  |
| Ever smoker | 1.01 [0.943-1.082] | 0.774 |
| **Clinical factors** |  |  |
| BMI |  |  |
| Under-weight | 1.138 [0.94-1.377] | 0.185 |
| Normal | reference |  |
| Over-weight | 1.073 [0.987-1.166] | 0.098 |
| Asthma=1 | 1.009 [0.943-1.08] | 0.791 |
| Diabetes=1 | 1.024 [0.952-1.102] | 0.525 |
| Dyslipidemia=1 | 0.976 [0.897-1.061] | 0.566 |
| CVD=1 | 1.055 [0.983-1.132] | 0.136 |
| History of AEs of COPD=1 | 1.113 [1.035-1.196] | 0.004 |

**Figure S1.** Comparison of ambient air pollutant exposure for participants

**Table S2.** Summary of adjusted ORs for the acute exacerbations of COPD associated with each unit increase in the concentrations of air pollutant

| Pair | pollutant | adjusted | OR [95% CI] | P |
| --- | --- | --- | --- | --- |
| NA | PM2.5 | NA | 1 [0.997-1.002] | 0.689 |
| NA | PM10 | NA | 1 [1-1.001] | 0.602 |
| NA | NO2 | NA | 0.996 [0.991-1] | 0.059 |
| NA | CO | NA | 0.902 [0.746-1.091] | 0.290 |
| NA | SO2 | NA | 0.997 [0.982-1.012] | 0.695 |
| NA | O3 | NA | 1.007 [1-1.013] | 0.046 |
| NO2_PM2.5 | NO2 | +PM2.5 | 0.995 [0.99-1] | 0.058 |
| PM2.5_NO2 | PM2.5 | +NO2 | 1.001 [0.998-1.003] | 0.653 |
| NO2_PM10 | NO2 | +PM10 | 0.996 [0.991-1] | 0.060 |
| PM10_NO2 | PM10 | +NO2 | 1 [1-1.001] | 0.606 |
| CO_PM2.5 | CO | +PM2.5 | 0.864 [0.664-1.125] | 0.280 |
| PM2.5_CO | PM2.5 | +CO | 1.001 [0.998-1.004] | 0.647 |
| CO_PM10 | CO | +PM10 | 0.864 [0.702-1.063] | 0.168 |
| PM10_CO | PM10 | +CO | 1 [1-1.001] | 0.306 |
| CO_NO2 | CO | +NO2 | 1.028 [0.8-1.321] | 0.827 |
| NO2_CO | NO2 | +CO | 0.995 [0.989-1.001] | 0.115 |
| SO2_PM2.5 | SO2 | +PM2.5 | 0.998 [0.978-1.019] | 0.875 |
| PM2.5_SO2 | PM2.5 | +SO2 | 1 [0.997-1.003] | 0.859 |
| SO2_PM10 | SO2 | +PM10 | 0.993 [0.975-1.011] | 0.420 |
| PM10_SO2 | PM10 | +SO2 | 1 [1-1.001] | 0.381 |
| SO2_NO2 | SO2 | +NO2 | 1.002 [0.986-1.018] | 0.776 |
| NO2_SO2 | NO2 | +SO2 | 0.995 [0.991-1] | 0.062 |
| SO2_CO | SO2 | +CO | 1.003 [0.984-1.023] | 0.730 |
| CO_SO2 | CO | +SO2 | 0.878 [0.688-1.121] | 0.298 |
| O3_PM2.5 | O3 | +PM2.5 | 1.009 [1.001-1.016] | 0.020 |
| PM2.5_O3 | PM2.5 | +O3 | 0.998 [0.996-1.001] | 0.206 |
| O3_PM10 | O3 | +PM10 | 1.008 [1-1.016] | 0.042 |
| PM10_O3 | PM10 | +O3 | 1 [0.999-1.001] | 0.512 |
| O3_NO2 | O3 | +NO2 | 1.006 [1-1.013] | 0.065 |
| NO2_O3 | NO2 | +O3 | 0.996 [0.992-1.001] | 0.084 |
| O3_CO | O3 | +CO | 1.007 [1-1.014] | 0.040 |
| CO_O3 | CO | +O3 | 0.893 [0.738-1.08] | 0.244 |
| O3_SO2 | O3 | +SO2 | 1.007 [1-1.013] | 0.048 |
| SO2_O3 | SO2 | +O3 | 0.998 [0.983-1.013] | 0.782 |

**Table S3.** Adjusted ORs for the acute exacerbations of COPD associated with each unit increase in the concentrations in air pollutants in stratified analyses

|  | OR [95% CI] | P | OR [95% CI] | P |
| --- | --- | --- | --- | --- |
|  | Gold grade 1 | | Gold grade 32 | |
| PM2.5 | 0.999 [0.997-1.002] | 0.686 | 1 [0.995-1.006] | 0.876 |
| PM10 | 1 [0.999-1.001] | 0.787 | 1.001 [0.999-1.002] | 0.500 |
| NO2 | 0.996 [0.992-1.001] | 0.161 | 0.994 [0.983-1.004] | 0.236 |
| CO | 0.929 [0.756-1.142] | 0.487 | 0.837 [0.491-1.429] | 0.517 |
| SO2 | 1 [0.983-1.016] | 0.953 | 0.997 [0.959-1.036] | 0.872 |
| O3 | 1.006 [0.999-1.014] | 0.107 | 1.011 [0.995-1.027] | 0.187 |
|  | Female | | Male | |
| PM2.5 | 1 [0.996-1.003] | 0.810 | 0.999 [0.997-1.002] | 0.633 |
| PM10 | 1 [0.999-1.001] | 0.455 | 1 [0.999-1.001] | 0.966 |
| NO2 | 0.994 [0.986-1.002] | 0.123 | 0.996 [0.99-1.001] | 0.138 |
| CO | 0.902 [0.66-1.234] | 0.520 | 0.888 [0.693-1.138] | 0.348 |
| SO2 | 0.988 [0.963-1.013] | 0.336 | 1.004 [0.984-1.023] | 0.717 |
| O3 | 1.004 [0.992-1.015] | 0.539 | 1.009 [1-1.017] | 0.046 |
|  | Age <= 65 | | Age > 65 | |
| PM2.5 | 0.999 [0.996-1.002] | 0.404 | 1 [0.997-1.003] | 0.964 |
| PM10 | 1 [0.999-1.001] | 0.710 | 1 [0.999-1.001] | 0.332 |
| NO2 | 0.995 [0.989-1.002] | 0.145 | 0.995 [0.989-1.001] | 0.134 |
| CO | 0.85 [0.651-1.111] | 0.236 | 0.918 [0.697-1.208] | 0.540 |
| SO2 | 0.99 [0.971-1.009] | 0.317 | 1.003 [0.979-1.028] | 0.817 |
| O3 | 1.002 [0.993-1.012] | 0.617 | 1.012 [1.002-1.021] | 0.014 |
|  | Uyghur | | Han | |
| PM2.5 | 1 [0.997-1.003] | 0.858 | 1 [0.996-1.005] | 0.838 |
| PM10 | 1 [0.999-1.001] | 0.741 | 1.001 [0.999-1.002] | 0.278 |
| NO2 | 0.997 [0.99-1.003] | 0.309 | 0.997 [0.99-1.005] | 0.467 |
| CO | 0.931 [0.723-1.198] | 0.577 | 1.004 [0.709-1.424] | 0.981 |
| SO2 | 0.99 [0.972-1.008] | 0.265 | 1.027 [0.997-1.058] | 0.075 |
| O3 | 1.003 [0.994-1.012] | 0.495 | 1.019 [1.007-1.032] | 0.003 |
|  | Rural | | Urban | |
| PM2.5 | 1 [0.997-1.003] | 0.999 | 0.999 [0.996-1.003] | 0.659 |
| PM10 | 1 [0.999-1.001] | 0.783 | 1 [0.999-1.001] | 0.666 |
| NO2 | 0.998 [0.991-1.005] | 0.610 | 0.995 [0.989-1.001] | 0.094 |
| CO | 0.996 [0.778-1.275] | 0.974 | 0.786 [0.585-1.056] | 0.111 |
| SO2 | 0.99 [0.973-1.008] | 0.293 | 1.008 [0.983-1.034] | 0.523 |
| O3 | 1.009 [0.999-1.019] | 0.074 | 1.006 [0.997-1.015] | 0.229 |
|  | Never-smoking | | Ever-smoking | |
| PM2.5 | 1 [0.997-1.002] | 0.744 | 0.999 [0.995-1.003] | 0.558 |
| PM10 | 1 [1-1.001] | 0.371 | 1 [0.999-1.001] | 0.552 |
| NO2 | 0.993 [0.987-0.999] | 0.018 | 0.999 [0.992-1.006] | 0.711 |
| CO | 0.839 [0.66-1.067] | 0.153 | 0.974 [0.702-1.353] | 0.877 |
| SO2 | 0.994 [0.975-1.013] | 0.523 | 1.003 [0.978-1.028] | 0.840 |
| O3 | 1.008 [0.999-1.017] | 0.069 | 1.005 [0.994-1.016] | 0.411 |
|  | AE prior year = No | | AE prior year = Yes | |
| PM2.5 | 1 [0.996-1.003] | 0.787 | 1 [0.997-1.003] | 0.849 |
| PM10 | 1 [0.999-1.001] | 0.640 | 1 [1-1.001] | 0.376 |
| NO2 | 0.999 [0.992-1.007] | 0.831 | 0.995 [0.989-1] | 0.063 |
| CO | 1.123 [0.815-1.546] | 0.480 | 0.843 [0.663-1.072] | 0.165 |
| SO2 | 1.009 [0.985-1.033] | 0.476 | 0.992 [0.973-1.011] | 0.412 |
| O3 | 1.003 [0.991-1.014] | 0.627 | 1.008 [1-1.017] | 0.048 |
|  | CVD = No | | CVD = Yes | |
| PM2.5 | 0.999 [0.996-1.002] | 0.590 | 0.999 [0.997-1.002] | 0.700 |
| PM10 | 1 [0.999-1.001] | 0.989 | 1 [0.999-1.001] | 0.701 |
| NO2 | 0.997 [0.99-1.004] | 0.349 | 0.995 [0.989-1.001] | 0.089 |
| CO | 0.854 [0.633-1.154] | 0.306 | 0.918 [0.715-1.178] | 0.501 |
| SO2 | 0.991 [0.97-1.012] | 0.407 | 1.001 [0.98-1.022] | 0.924 |
| O3 | 1.006 [0.995-1.017] | 0.304 | 1.007 [0.998-1.016] | 0.114 |
|  | Asthma = No | | Asthma = Yes | |
| PM2.5 | 1 [0.997-1.004] | 0.874 | 0.999 [0.996-1.002] | 0.521 |
| PM10 | 1.001 [1-1.002] | 0.196 | 1 [0.999-1.001] | 0.721 |
| NO2 | 0.994 [0.988-1.001] | 0.100 | 0.996 [0.99-1.002] | 0.234 |
| CO | 0.828 [0.622-1.103] | 0.198 | 0.963 [0.745-1.243] | 0.770 |
| SO2 | 1.002 [0.979-1.026] | 0.861 | 0.993 [0.974-1.012] | 0.464 |
| O3 | 1.014 [1.004-1.025] | 0.009 | 1.002 [0.994-1.011] | 0.628 |
|  | Season of baseline = Spring | | Season of baseline = Winter | |
| PM2.5 | 1 [0.997-1.004] | 0.854 | 0.999 [0.996-1.002] | 0.470 |
| PM10 | 1 [0.999-1.001] | 0.494 | 1 [0.999-1.001] | 0.881 |
| NO2 | 0.997 [0.991-1.004] | 0.432 | 0.994 [0.988-1] | 0.038 |
| CO | 0.942 [0.701-1.265] | 0.690 | 0.88 [0.678-1.142] | 0.337 |
| SO2 | 1.008 [0.986-1.031] | 0.475 | 0.989 [0.969-1.01] | 0.311 |
| O3 | 1.006 [0.994-1.018] | 0.349 | 1.006 [0.997-1.015] | 0.176 |
